# Comparative “Golgi” Proteome Study of *Lolium multiflorum* and *Populus trichocarpa*

**DOI:** 10.3390/proteomes4030023

**Published:** 2016-07-20

**Authors:** Kristina L. Ford, Tony Chin, Vaibhav Srivastava, Wei Zeng, Monika S. Doblin, Vincent Bulone, Antony Bacic

**Affiliations:** 1 Australian Research Council Centre of Excellence in Plant Cell Walls, School of BioSciences, The University of Melbourne, Victoria 3010, Australia; kferg@unimelb.edu.au (K.L.F.); tonyccm@gmail.com (T.C.); zengw@unimelb.edu.au (W.Z.); msdoblin@unimelb.edu.au (M.S.D.); 2 Division of Glycoscience, School of Biotechnology, Royal Institute of Technology (KTH), AlbaNova University Centre, 106 91 Stockholm, Sweden; vasri@kth.se (V.S.); bulone@kth.se (V.B.); 3 Australian Research Council Centre of Excellence in Plant Cell Walls, School of Agriculture, Food and Wine, University of Adelaide, Waite Campus, Urrbrae, SA 5064, Australia

**Keywords:** Golgi apparatus, sub-cellular fractionation, subcellular proteomics, quantitative proteomics

## Abstract

The Golgi apparatus (GA) is a crucial organelle in the biosynthesis of non-cellulosic polysaccharides, glycoproteins and proteoglycans that are primarily destined for secretion to the cell surface (plasma membrane, cell wall and apoplast). Only a small proportion of the proteins involved in these processes have been identified in plants, with the majority of their functions still unknown. The availability of a GA proteome would greatly assist plant biochemists, cell and molecular biologists in determining the precise function of the cell wall-related proteins. There has been some progress towards defining the GA proteome in the model plant system *Arabidopsis thaliana*, yet in commercially important species, such as either the cereals or woody species there has been relatively less progress. In this study, we applied discontinuous sucrose gradient centrifugation to partially enrich GA from suspension cell cultures (SCCs) and combined this with stable isotope labelling (iTRAQ) to determine protein sub-cellular locations. Results from a representative grass species, Italian ryegrass (*Lolium multiflorum*) and a dicot species, black cottonwood (*Populus trichocarpa*) are compared. The results confirm that membrane fractionation approaches that provide effective GA-enriched fractions for proteomic analyses in *Arabidopsis* are much less effective in the species examined here and highlight the complexity of the GA, both within and between species.

## 1. Introduction

The Golgi apparatus (GA) is the cell’s “engine room” for glycosylation and hence plays an important role in the biosynthesis of non-cellulosic polysaccharides, glycoproteins and proteoglycans, the major macromolecular components of the plant cell surface. The GA is vital for plant growth and development, as well as for responses to abiotic and biotic stress [1]. It is an important component of a very dynamic membranous secretory pathway, which contributes significantly to the challenges of defining the GA proteome [2]. While a number of proteins are resident to the GA, many are transiently associated with the organelle and exert their function elsewhere in the cell. This is the case for all proteins that transit through the GA during secretion to their final destination at either the vacuole or the cell surface. The GA is embedded in a matrix and is also tightly associated with the endoplasmic reticulum (ER), although the way these two organelles are associated is still disputed [3]. Undoubtedly, the similarity of the structural properties of the GA and ER makes them difficult to separate 

GA enrichment was successfully used to study enzymes involved in cell wall biosynthesis [4,5] well before attempts were made to define the GA proteome. There has been some progress towards mapping the GA proteome in the model plant system *Arabidopsis* [6,7,8,9,10]. However, it has been estimated that out of the 2239 ± 465 predicted GA proteins only 20% have been experimentally identified thus far by combining all published *Arabidopsis* GA proteomes [2]. In contrast, there has been little progress in experimentally defining the GA proteome of commercially important plant species, such as agricultural crops and trees. For example, a total of only 106 and 32 GA proteins have been identified in rice [11,12] and the conifer *Pinus radiata* [13,14], respectively.

One of the difficulties faced in studying the GA proteome is that there is not one universal purification/fractionation method that works equally for all plant systems. For example, the Free Flow Electrophoresis (FFE) method used in *Arabidopsis* to produce a GA-enriched fraction of 80% purity [9], performed poorly when applied to *Pinus radiata* [13], with only 5%–10% of the identified proteins predicted to belong to the GA. Furthermore, the FFE method involves specialized and expensive instrumentation that requires challenging optimization and as a consequence has not been widely adopted. The most straightforward and widely adopted method is sucrose gradient centrifugation even though significant contamination from other organelles is unavoidable. An extension of the sucrose gradient centrifugation technique is the Localization of Organelle Proteins by Isotope Tagging (LOPIT) method which makes the assumption that proteins from the same organelle fractionate similarly on a density gradient; it is typically combined with the use of stable isotope tagging and predictive bioinformatic tools to identify potential GA-resident proteins [6].

Here we have chosen to study suspension cell cultures (SCCs) as they provide a ready source of a homogenous single cell type and overcome the difficult extraction of proteins from resilient woody tissues. Two different plant systems were used, i.e., *L. multiflorum* and *Populus trichocarpa*, a commelinid monocot and a woody dicot, respectively, that differ markedly in the polysaccharide composition of their cell walls. The *L. multiflorum* cell wall is rich in β-1,3;1,4-glucans (mixed linked glucans (MLGs)) and hetero (arabino) xylans [15], while the *P. trichocarpa* cell wall lacks MLGs but contains other cell wall carbohydrates typical of eudicots, i.e., xyloglucans and pectins rather than heteroxylans. We have used a similar but somewhat simpler approach to LOPIT [6,7], with discontinuous sucrose gradient centrifugation and stable isotope labelling (iTRAQ) to fractionate the sub-cellular membranes and determine protein identity and sub-cellular locations.

## 2. Materials and Methods

### 2.1. Plant Material

All chemicals were from Sigma-Aldrich (St. Louis, MO, USA), unless specified. *L. multiflorum* and *P. trichocarpa* SCCs were grown as described earlier [16,17].

### 2.2. Tissue Extraction

Seven-day-old *L. multiflorum* and *P. trichocarpa* SCCs were used for protein extraction. SCCs (100 g) were ground in a mortar and pestle with an extraction buffer consisting of 5% (*v*/*v*) 0.1 M MES buffer pH 6.5; 5% (*w*/*v*) sucrose; 1 mM EDTA; 2 mM EGTA; Roche EDTA-free protease inhibitor cocktail (1 tablet per 50 mL of buffer; Roche Applied Science, Penzburg, Germany) at 4 °C and filtered through two layers of Miracloth (Calbiochem, San Deigo, CA, USA). The filtrates were centrifuged in a Heraeus Multifuge 3 SR (ThermoFisher Scientific, Waltham, MA, USA) at 1000× *g* for 10 min at 4 °C to remove cell wall debris. 

### 2.3. Microsomal Membrane Preparation

A portion of the tissue extract was used to prepare the microsomal membrane (MM) fractions by centrifuging the previous filtrates at 100,000× *g* for 60 min in an Optima L-80 XP ultracentrifuge (Beckman Coulter, Brea, CA, USA) and re-suspending the resulting pellet in 0.1 M MES pH 6.5, 1 mM EDTA and 2 mM EGTA.

### 2.4. Membrane Fractionation

These membrane extracts were layered onto discontinuous gradients consisting of 5 mL each of 20%, 35% and 50% sucrose (*w*/*v*), prepared in 0.1 M MES buffer pH 6.5; 5% (*w*/*v*) sucrose; 1 mM EDTA; 2 mM EGTA. The discontinuous gradients were centrifuged at 100,000× *g* for 60 min at 4 °C. Membrane samples were collected from the supernatant/20% (*w*/*v*) sucrose interface (S1 fraction), 20% (*w*/*v*)/35% (*w*/*v*) sucrose interface (S2 fraction) and 35% (*w*/*v*)/50% (*w*/*v*) sucrose interface (S3 fraction). Each of the fractions was diluted at least 5 times with 0.1 M MES buffer pH 6.5; 5% (*w*/*v*) sucrose; 1 mM EDTA; 2 mM EGTA and pelleted by centrifugation at 50,000× *g* for 45 min at 4 °C. The pellets were washed twice with 0.1 M MES, pH 6.5; 5% (*w*/*v*) sucrose; 1 mM EDTA; 2 mM EGTA and re-suspended in the same mixture devoid of sucrose. Protein concentrations were determined using the BCA protein assay kit (ThermoFisher Scientific).

### 2.5. Enzyme Marker Assays

The latent inosine diphosphatase (IDPase) activity was used as a marker for the GA, the vanadate-sensitive Mg^2+^-dependent ATPase activity was used as a plasma membrane (PM) marker and the nitrate-sensitive ATPase activity was used as a vacuolar marker as described previously [18,19]. The mitochondrial cytochrome-*c* oxidase (Sigma-Aldrich, St. Louis, MO, USA), and the endoplasmic reticulum (ER) cytochrome-*c* reductase (Sigma) assays, were performed following the manufacturer’s instructions.

### 2.6. SDS-PAGE and Western Blot Analysis

Protein fractions (10 μg of each) were separated using NuPAGE Novex 4%–12% Bis-Tris Protein SDS-PAGE Gels (ThermoFisher Scientific) and proteins transferred onto nitrocellulose membranes using the iBLOT 2 dry blot system (Life Technologies, Carlsbad, CA, USA) according to the manufacturer’s instructions. Western blotting was performed using the SNAP2 blot system (Merck Millipore, Billerica, MA, USA). Membranes were probed with 1:1000 dilutions of antibodies directed against the following proteins: reversibly glycosylated protein 1 (α-RGP 1) from pea (kindly provided by Kanwarpal Dhugga, Dupont Pioneer, Aurelia, IA, USA) as a marker for the GA, the PM H^+^-ATPase (Agrisera, Vännäs, Sweden) as a PM marker, cytochrome-*c* oxidase subunit II (anti-COX II) (Agrisera) as a marker for mitochondria (MT), luminal-binding protein (anti-BiP) (Agrisera) as a marker for ER and the vanadate-sensitive ATPase epsilon subunit (V-ATPase) (Agrisera) as a vacuole marker. Primary antibodies were detected with goat anti-rabbit IgG secondary antibody (1:2000 dilution) conjugated to Pierce horseradish peroxidase (ThermoFisher Scientific) and SuperSignal West Femto Maximum Sensitivity chemiluminescent substrate (ThermoFisher Scientific), then digitally captured using a Chemi-Doc MP imager (Bio-Rad, Hercules, CA, USA) and analyzed using the Image Lab software version 4.1 (Bio-Rad).

### 2.7. Protein Digestion and iTRAQ Labelling

**Figure 1:**
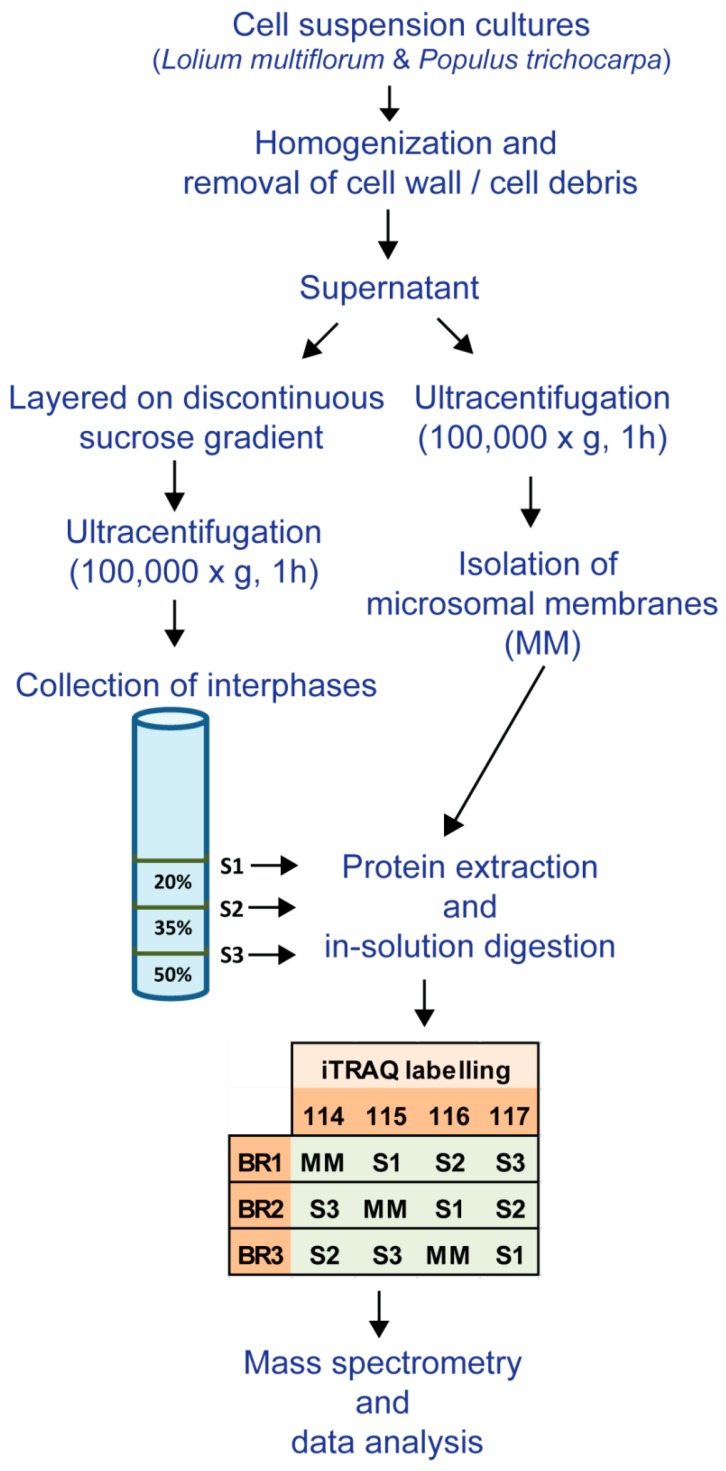
Experimental workflow used for the enrichment of microsomal membranes (MMs), the subcellular fractions (S1, S2 and S3) and the iTraq labelling of the biological replicates (BR1, BR2 and BR3) for the different membrane fractions from *L. multiflorum* and *P. trichocarpa* suspension cell cultures (SCCs).

Proteins were solubilized in 6 M urea; 250 mM triethylammonium bicarbonate (pH 8.5), reduced with 5 mM *tris-*(2-carboxyethyl) phosphine (TCEP) at 60 °C for 1 h, and alkylated with 10 mM methylmethane thiosulphate (MMTS) for 10 min at room temperature (RT). Samples were diluted to 1 M urea with water and proteins were digested with trypsin (4 μg; 1:25 *w*/*w*, sequencing grade, Promega, Madison, WI, USA) for 16 h at 37 °C. Proteolysis was stopped with the addition of 10 μL neat formic acid and peptide solutions were desalted using a Sep-Pak C18 Plus Short cartridge (Waters, Milford, MA, USA) as described previously [20]. Samples were concentrated under vacuum to approximately 10 μL and TEAB was added to each tube to reach a final volume of 30 μL. Peptides were labeled with iTRAQ 4plex reagent (Sciex, Framingham, MA, USA) following the manufacturer’s instructions. A separate set of iTRAQ tags were used for each biological replicate and labelled as shown in Figure 1. 

### 2.8. Non-labelled Protein Hydrolysis

Three biological replicates of the *L. multiflorum* S1 fraction and 2 biological replicates of the S1 *P. trichocarpa* were re-suspended in 6 M urea; 100 mM ammonium bicarbonate and reduced with 10 mM DTT at 60 °C for 1 h. The samples were allowed to cool and then alkylated with 50 mM iodoacetamide for 45 min at RT. The samples were diluted to 1 M urea with 100 mM ammonium bicarbonate and hydrolyzed with trypsin (4 μg; 1:25 *w*/*w*, sequencing grade, Promega) for 16 h at 37 °C. Proteolysis was stopped with 1 μL of neat formic acid and the sample desalted using a Sep-Pak C18 Plus Short cartridge (Waters) as described previously [20]. Samples were concentrated under vacuum to approximately 100 μL. 

### 2.9. Peptide Fractionation and Mass Spectrometry

iTRAQ and the *L. multiflorum* S1 samples were fractionated by strong cation exchange (SCX) chromatography and fractions analyzed by LC-MS/MS as described in Ford et al. [20]. Peptides (1 μg) from the *P. trichocarpa* S1 fraction were analyzed on a Q Exactive Plus mass spectrometer (Thermo Scientific) coupled to an Ultimate 3000 RSLC nanosystem (Dionex, Sunnyvale, CA, USA). The nanoLC system was equipped with an Acclaim Pepmap nano-trap column (Dionex) and an Acclaim Pepmap analytical column (Dionex), operating at a flow rate of 3 μL·min^−1^ with a 90 min gradient of 3%–80% (*v*/*v*) acetonitrile containing 0.1% formic acid. The Q Exactive Plus mass spectrometer was operated in positive mode, with the spray voltage set to 1800 kV, S-lens RF level at 50 and heated capillary at 250 °C. Peptides were fragmented using normalized collision energy of 35 and activation time of 0.1 ms in the data-dependent mode, whereby the top 10 ions between 400 and 1600 *m*/*z* with a charge state between 2^+^ and 5^+^ were selected for MS/MS.

### 2.10. Protein Identification

Mass spectra obtained from the *L. multiflorum* SCCs were searched against an in-house database for protein predictions from a *L. multiflorum* SCC transcriptome and the *Lolium perenne* V1 protein database [21]. Data from the *P. trichocarpa* samples were searched against the Populus protein database V3 [22] using ProteinPilot version 4.5 (Sciex) with the following parameters: Sample type: iTRAQ 4plex (Peptide labelled); Cys Alkylation: MMTS; Digestion: Trypsin; Search Effort: Thorough ID. Peak lists generated by ProteinPilot were exported and used to search against the same databases using MASCOT version 2.4 (Matrix Science, London, UK). The MASCOT parameters were: Enzyme: Trypsin; Fixed modifications: iTRAQ 4plex (N-term), iTRAQ 4plex (K), Methylthiol (C); MS peptide tolerance: 10 ppm; MS/MS tolerance: 0.15 Da; Number of missed cleavages: up to 1. The peptide list from MASCOT was generated with a *p* < 0.05. The peptide summary report was exported from ProteinPilot and filtered by confidence level according to the local 5% false discovery rate reported by ProteinPilot. Peptides found using both search algorithms were selected for further analysis. Peptides were used to search the databases (in the case of *L. multiflorum* the databases were combined into one) using KNIME [23] to find all proteins containing any of the peptides. The list was then reduced to a minimum set of proteins by selecting only those that contained two or more peptides; I and L were treated as the same amino acid. Where 2 or more proteins had the same set of peptides matching, the protein with the longest sequence was selected. 

The non-labelled spectra from the S1 protein profiling experiment were analyzed similarly to the iTRAQ spectra except for the following ProteinPilot parameters: Sample type: Identification; Cys Alkylation: Iodoacetamide; Digestion: Trypsin; Search Effort: Thorough ID. Peak lists generated by ProteinPilot were exported and used to search against the same databases using MASCOT version 2.4 (Matrix Science). The MASCOT parameters were: Enzyme: Trypsin; Fixed modifications: carbamidomethyl (C); MS peptide tolerance: 10 ppm; MS/MS tolerance: 0.15 Da; Number of missed cleavages: up to 1.

BLAST analyses were performed on the identified proteins against the TAIR10 [24] database and the *Oryza sativa* subsp. japonica Uniprot reference proteome (downloaded November 2015) [25]. *Arabidopsis* proteins with the highest similarity were used to search SUBA3 [26] for protein sub-cellular location based on the experimental data rather than the predicted locations and the Carbohydrate-Active Enzymes database (CAZy) [27] for possible cell wall-associated proteins. Sequences from the *P. trichocarpa* and *O. sativa* proteins with the highest similarity to the *L. multiflorum* sequence were used for analysis with SignalP 4.0 [28] to determine the possible occurrence of a signal peptide.

### 2.11. Relative Quantification

The reporter ion peak areas for the peptides identified above were imported into KNIME [23] from the peptide summary report generated in ProteinPilot and used for relative quantitation as applied in Ford et al. [20], with some modifications. Briefly, any peptide with a reporter ion peak area of less than 20 was removed from quantification. Only unique peptides and peptides with a charge state of either 2^+^ or 3^+^ were used for quantification. Each peptide signal was then normalized by the sum of the corresponding channel intensities (114, 115, 116 and 117). Peptides were ignored when the normalized peptide value was more than 2 SD units from the calculated mean of the protein the peptide matched to. The mean was then calculated for proteins with two or more peptides that fulfilled the above criteria. A change was considered significant if it was greater than 1.3-fold, based on a 1% FDR calculated from a same-same iTRAQ control experiment (Table S1).

Proteins were clustered using the *k-*means clustering node in KNIME [23] with *k* set to 13.

## 3. Results

### 3.1. Enrichment of Microsomal Membranes

**Figure 2:**
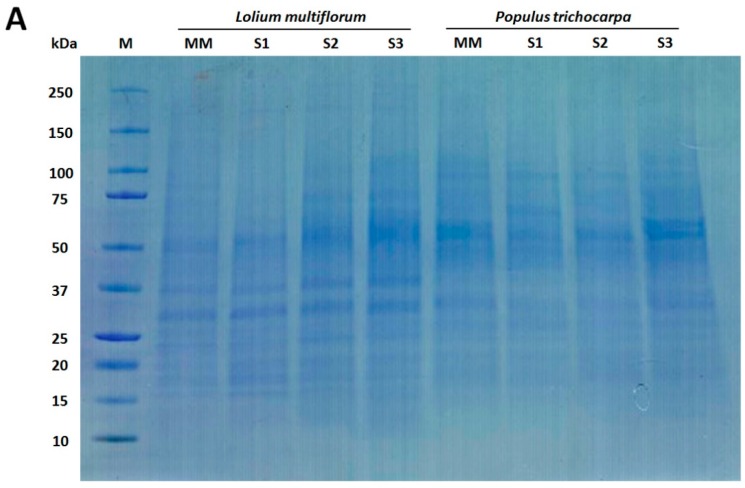

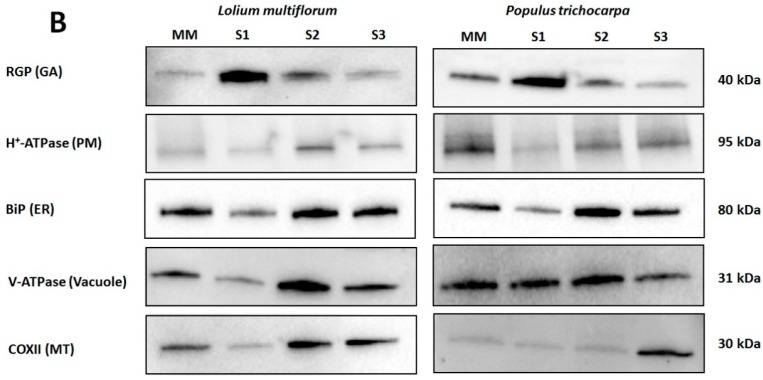
Western blot analysis of *Lolium multiflorum* and *Populus trichocarpa* suspension cell culture (SCC) membrane fractions. Fractions (10 μg of total protein each) from MM, S1, S2, and S3 were separated by SDS-PAGE. (**A**) Coomassie blue staining of the total proteins separated on SDS-PAGE demonstrating the equal protein loading across the membrane fractions, (**B**) Western blots performed with anti-RGP (GA marker), anti-H^+^-ATPase (PM marker), anti-BiP (ER marker), anti-V-ATPase (vacuole marker), and anti-COX II (MT marker) antibodies. The molecular masses of the respective marker proteins are indicated on the right.

Enrichment of microsomal membranes (MMs) was assessed by enzyme marker assays and Western blot analysis using antibodies to proteins known to be located in particular membrane types. For *L. multiflorum* the S1 fraction showed an increase in IDPase acitivity (GA) (Table 1) consistent with an increase of α-RGP 1 (GA) signal compared to all other fractions (Figure 2B). Decreases in both the nitrate- and vanadate-sensitive ATPases (vacuole and PM, respectively) as well as the cytochrome-*c* oxidase assay (MT) in S1 compared to all other fractions (Table 1) are consistent with the decrease in H^+^-ATPase (PM), COX II (MT) and the V-ATPase (vacuole) markers, respectively. The ER markers, cytochrome-*c* reductase activity (Table 1) and BiP (Figure 2B) showed a small decrease in S1 compared to all other fractions. Thus, for *L. multiflorum* the S1 fraction was chosen for proteomic analyses as this was assumed to be the most “GA-enriched” fraction by both the Western blot antibody marker distributions and the marker enzyme activity with a calculated 1.7-fold enrichment for the latter.

In *P. trichocarpa* the S1 fraction showed an increased α-RGP 1 (GA) signal compared to the MM, S2 and S3 fractions as well as decreases in H^+^-ATPase (PM), COX II (MT) and the BiP2 (ER) markers (Figure 2B), consistent with an enrichment of GA. There appears to be considerable vacuolar contamination with little variation in the V-ATPase (vacuole) marker (Figure 2B) in the S1 compared to the S2 and MM fractions.

### 3.2. Mass Spectrometric Analyses

#### 3.2.1. Protein Profiling of the Non-labelled GA-Enriched Fractions from *L. multiflorum* and *P. trichocarpa*

The *L. multiflorum* S1 fraction contained 835 proteins that were identified with at least 2 peptides, in at least 2 biological replicates (Table S2) whereas the *P. trichocarpa* counterpart contained 1236 proteins identified with at least 2 peptides, in 2 biological replicates (Table S3). BLAST analysis of the *L. multiflorum* proteins against the *Arabidopsis* database revealed that 79 of these proteins are known to reside in the GA, 127 proteins have been identified as GA-localized in previous proteomic analysis [6,7,8,9,10,11,29] and 35 proteins present significant levels of similarity to proteins in the CAZy database [27]. The *O. sativa* sequence with the highest similarity to each *L. multiflorum* sequence was used for signal peptide prediction using SignalP as more complete sequences were needed. There were 79 proteins identified with a predicted signal peptide, indicating that they are destined for delivery to post-Golgi membrane compartments downstream in the secretion pathway. In total, this provided a list of 211 (25%) of the identified proteins conceivably either residing or transiting through the GA. The same analysis of the *P. trichocarpa* proteins revealed 69 proteins known to reside within the GA, 104 proteins identified as GA-localized in previous proteomic analysis [6,7,8,9,10,11,29], 33 proteins with similarities to proteins in the CAZy database and 96 proteins with a predicted signal peptide. In total, 227 (18%) proteins were identified that likely reside or transit through the GA. Of the remaining *L. multiflorum* proteins there were 81 (10%) known MT proteins, 34 (4%) PM proteins, 37 (4%) ER proteins, 42 (5%) cytoplasmic proteins (CP), 29 (3%) nuclear proteins, 27 (3%) plastid proteins, 8 (1%) vacuolar proteins and 375 (45%) proteins without a known sub-cellular location. Comparatively for the remaining *P. trichocarpa* proteins, 80 (6%) were known MT proteins, 68 (6%) PM proteins, 11 (1%) ER proteins, 137 (11%) cytoplasmic proteins (CP), 26 (2%) nuclear proteins, 47 (4%) plastid proteins, 23 (2%) vacuolar proteins and 628 (51%) proteins without a known location.

Amongst the GA proteins identified in the *L. multiflorum* S1 fraction was the GA marker protein RGP1 (Table S2). Even though RGP1 was not identified in *P. trichocarpa,* RGP2 and RGP3, two proteins from the same family, were identified in this species (Table S3). In both the grass and tree cell samples, the more abundant GA proteins were the endomembrane 70 (EMP70) proteins and *S*-adenosyl-l-methionine-dependent methyltransferases (SAM). Enzymes active on nucleotides or involved in nucleotide-sugar interconversion that were reported in other GA proteomes were also present in both species, such as apyrase 1, UDP-d-glucose/UDP-d-galactose 4 epimerase (UGE1) and UDP-d-glucose pyrophosphorylase 2 (UGP2) in *L. multiflorum* and UGP2 and an uncharacterized nucleotide-diphospho-sugar transferase in *P. trichocarpa*. Proteins expected to reside in the *trans-*Golgi network (TGN) were well represented, with the presence of RAB GTPases and vacuolar sorting receptors (VSR) in both species, with the addition of the YIP1, syntaxins and transport protein particle (TRAPP) proteins in *L. multiflorum* (Table S2) and the *N*-ethylmaleimide-sensitive factor attachment protein receptors (SNARE) and vesicle-associated membrane proteins (VAMP) in *P. trichocarpa* (Table S3). Proteins expected to be more loosely associated with the GA and identified within the S1 fraction in both species were the coatomer complex proteins, with the addition of tether proteins in *L. multiflorum*, such as the conserved oligomeric Golgi complex (COG) and golgin candidate 6 (GC6).

A more noticeable difference between the two species was observed in the glycosyltransferases (GTs) identified in the GA-enriched fractions. In *L. multiflorum* there were 5 proteins identified from the GT2 family involved in cell wall biosynthesis, i.e., cellulose synthase-like F6 (CSLF6) involved in the synthesis of MLG [30], cellulose synthases involved in primary cell wall biosynthesis (CESAs 1, 3 and 6) and a cellulose synthase-like D (CSLD) possibly involved in (1-4)-β-glucan [31] or mannan [32] activity. Five glucan synthase-like (GSL) proteins predicted to be responsible for callose biosynthesis [33] were identified from the GT48 family. Other GTs present in the *L. multiforum* samples were 3 GTs from the GT61 family and 1 from the GT47 family, both families have been implicated in xylan biosynthesis [34,35], a GT29 protein, possibly involved in pectin biosynthesis [36], the UDP-glucose: glycoprotein glycosyltransferase (EBS1) involved in protein folding and an uncharacterized GT with a domain of unknown function (DUF707) [27]. In *P. trichocarpa* neither CESA nor GSL proteins were observed, or any other GT involved in cell wall biosynthesis, possibly due to the lower GA-enrichment in the samples from this species combined with an expected low abundance of GTs. The only similarity between the lists of GTs observed in the samples from the two species was the presence of the dolichyl-diphospho-oligosaccharide protein glycosyltransferase (DGL1) involved in protein *N*-glycosylation [37] from the GT2 family and trehalose phosphatase/synthase 7 (ATTPS7) involved in secondary metabolism [38] from the GT20 family, both contaminants from the ER and the CP, respectively. In addition, a different set of GTs was observed in the samples from *P. trichocarpa*, which contained 3 GT1 family members, UDP-glycosyltransferase 71B1 (UGT71B1), UDP-glycosyltransferase 73B4 (UGT73B4) and an uncharacterized UDP-glycosyltransferase, as well as the UDP-glycosyltransferase/trehalose phosphatase (ATTPS6) from the GT20 family. These 4 GTs are most likely involved in secondary metabolism rather than cell wall biosynthesis. 

#### 3.2.2. iTRAQ Analysis of the *L. multiflorum* and *P. trichocarpa* GA-enriched Fractions

**Figure 3:**
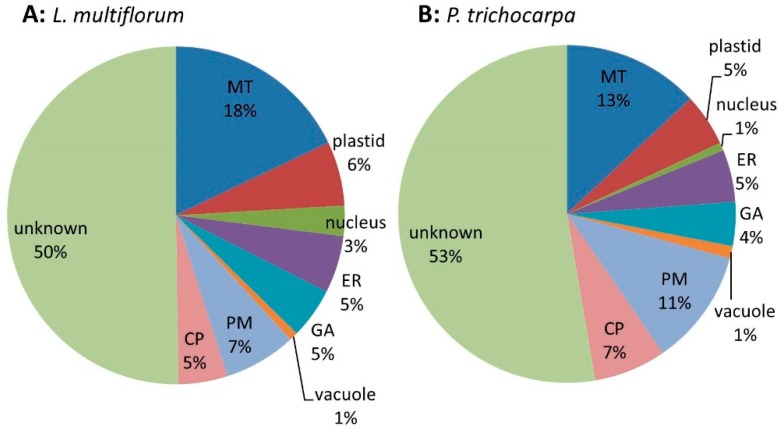
Pie chart of the expected sub-cellular locations of (**A**) the 619 proteins from *L. multiflorum* and (**B**) the 260 proteins from *P. trichocarpa* from which quantitative data were obtained. The pie charts show a similar distribution of proteins in the different sub-cellular compartments, i.e., mitochondria (MT), endoplasmic reticulum (ER), Golgi apparatus (GA), plasma membrane (PM), vacuole and cytoplasmic (CP).

Samples prepared from three biological replicates were fractionated into MM, S1, S2 and S3 and their protein composition was compared using the iTRAQ technology. Quantitative data were obtained for a total of 619 proteins (Table S4) across all 3 biological replicates from *L. multiflorum*. These proteins were assigned putative sub-cellular locations based on experimental data of *Arabidopsis* proteins (SUBA3) with the highest similarity to the *Lolium* sequences. Proteins that have been shown to reside in multiple locations were assigned as ‘unknown’. Of the 619 proteins 18% were predicted to be located in MT, 6% in plastids, 3% in nucleus, 5% in the ER, 5% in GA, 1% in the vacuole, 7% in the PM, 5% in the cytosoluble phase (CP) and the remaining 50% as unknown (Figure 3A). Quantitative data were obtained from 260 proteins in the *P. trichocarpa* samples (Table S5) with similar proportions as for *L. multiflorum*. These proteins are predicted to belong to the various subcellular organelles, with 13% assigned to MT, 5% to plastids, 1% to nucleus, 5% to ER, 4% to GA, 1% to vacuole, 11% to PM, 7% to CP and the remaining 53% as unknown (Figure 3B). 

**Figure 4:**
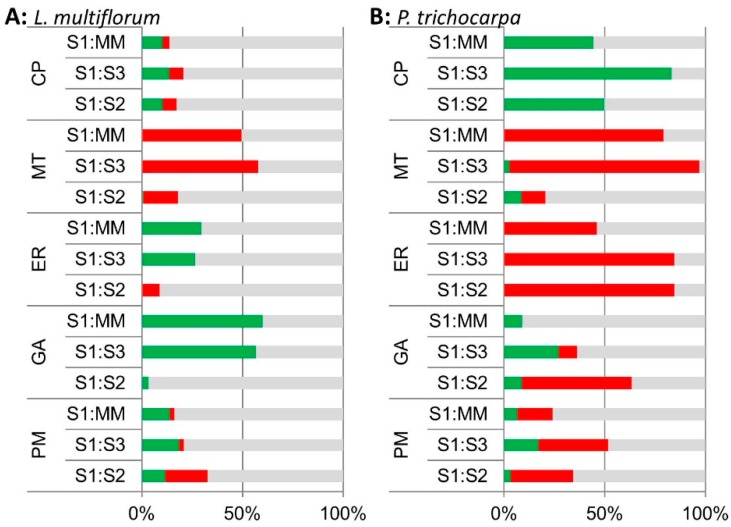
Bar chart of the percentage of proteins in the S1:MM, S1:S3 and S1:S2 iTRAQ ratios that are significantly enriched (green), depleted (red) and unchanged (grey) across all 3 biological replicates based on their expected sub-cellular locations; cytoplasmic proteins (CP), mitochondria (MT), endoplasmic reticulum (ER), Golgi (GA) and plasma membrane (PM) locations in (**A**) *L. multiflorum* and (**B**) *P. trichocarpa* samples based on iTRAQ analyses.

A comparison of the percentage of proteins changing in their iTRAQ ratios in the different predicted sub-cellular locations identified significant increases in GA and ER proteins in S1 compared to MM (S1:MM) and S1:S3 in the *L. multiflorum* samples (Figure 4A). Surprisingly, the *P. trichocarpa* samples had a different pattern, with most significant increases occurring for CP proteins in S1:S3, S1:S2 and S1:MM, and only some increases in GA proteins in S1:MM and S1:S3, most of which decreased in S1:S2 (Figure 4B). Samples from both plant species showed a decrease in MT protein in S1 relative to MM, S2 and S3.

Of the thirty proteins that were assigned as GA-localized in *L. multiflorum*, only one, the vacuolar sorting receptor homolog 1, was significantly increased in S1:S2 (Table S4). The GA proteins are mostly enriched in S1:S3 and S1:MM (Figure 4A). There were a total of 79 proteins significantly enriched across all 3 biological replicates in S1:S3 and S1:MM. Over half (17) of the GA proteins were significantly enriched in both S1:S3 and S1:MM, together with 3 CP proteins, 8 ER proteins, 6 PM proteins, 2 nuclear proteins, 2 vacuolar proteins, 1 plastid protein and 40 proteins with an unclear, hence unassigned, sub-cellular location. Collectively, approximately 30% of these proteins (24) have previously been observed as GA-localized in other proteomics experiments [6,7,8,9,10,29]. These included GA proteins identified in the non-labelled protein profiling of the S1 fraction, EMP70, VSR, apyrase and nucleotide-sugar interconversion proteins.

**Figure 5:**
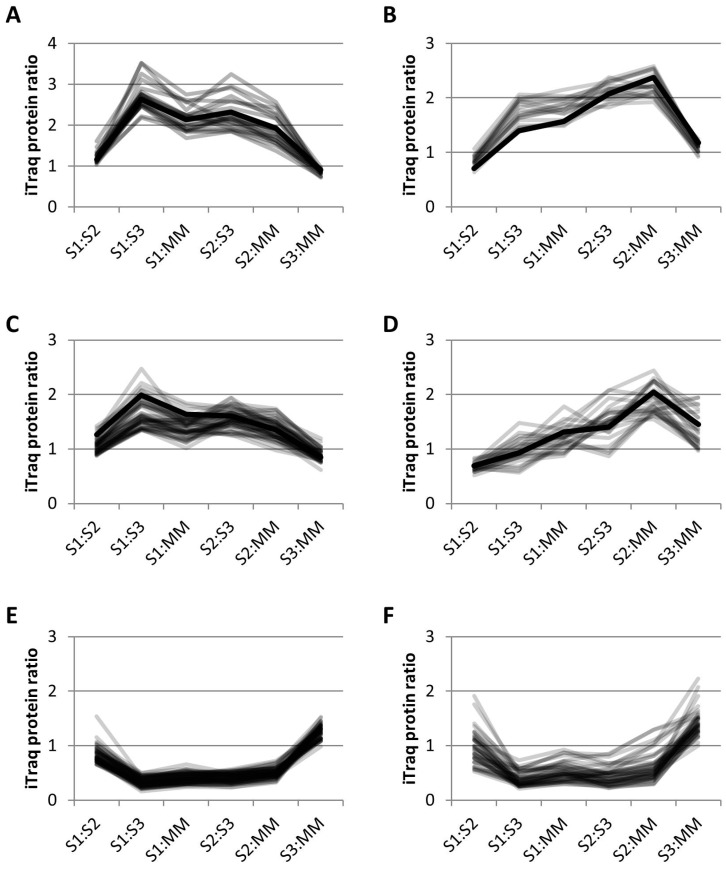
Relative abundance profiles using *k-*means clustering of iTRAQ data. (**A**) *L. multiflorum* cluster 13; 22 proposed GA proteins in grey and the profile of apyrase, a well characterized GA protein in black; (**B**) *L. multiflorum* cluster 12; 13 proposed ER proteins in grey and the profile of the ER dolichyl-diphosphooligosaccharide glycosyltransferase subunit 2 (DGL2) in black; (**C**) *L. multiflorum* cluster 9; 49 proposed TGN proteins in grey and known TGN protein, vacuolar sorting protein 45 (VSPS45) in black, (**D**) *P. trichocarpa* cluster 9; 25 proposed TGN proteins in grey and known TGN protein, vacuolar ATPase subunit D (VATD) in black, (**E**) *L. multiflorum* cluster 11; 61 proposed MT proteins, and (**F**) *P. trichocarpa* cluster 11; 44 proposed MT proteins.

Clustering using *k-*means (see Materials and Methods) separated the iTRAQ data into 13 different clusters. Of the 24 proteins previously identified as GA-localized, 13 clustered together using *k*-means clustering, along with 9 other proteins in cluster 13 (Figure 5A). Sixty-eight percent of the proteins in this group have been identified in previous proteomics studies (Table 2). In this cluster, there were 22 proteins that included 13 GA proteins, 2 vacuolar proteins and 7 unassigned proteins. There were 4 EMP70 family proteins in this cluster that are reportedly the most abundant GA proteins [8]. Although the monosaccharide transporter is labelled as a tonoplast protein in the TAIR10 *Arabidopsis* database, it contains a KXD/E motif at the C-terminus, indicative of GA localization [39], as observed previously [9]. There were 2 nucleotide-sugar biosynthetic proteins, i.e., a UDP-glucuronic acid decarboxylase and the UDP-arabinose 4-epimerase 1, as well as a putative sugar phosphate/phosphate translocator. The *L. multiflorum* protein p6229 contains an *O*-fucosyltransferase (FucT)-like domain and has some homology to an unclassified *Arabidopsis* GT. The apyrase 1 protein is within this cluster and has previously been localized to the GA both by proteomics and microscopy [9]. Thus, the 22 proteins in this cluster could all conceivably be GA proteins.

Thirty-one percent of the proteins in cluster 12 (Figure 5B, Table S6) have previously been identified in GA proteomes. This cluster contained 7 out of the 8 ER proteins that are significantly enriched in S1:S3 and S1:MM (Figure 4A), as well as 6 other proteins expected to be ER-localized, together with 1 plastid and 5 unassigned proteins. Included in this cluster was DGL2, an essential subunit of the *N*-oligosaccharyltransferase (OST) complex which catalyses the transfer of a high mannose oligosaccharide from a lipid-linked oligosaccharide donor to a nascent polypeptide chain; an evolutionarily conserved and a well characterized process that occurs in the ER [40]. The plastid protein has been observed in previous proteomics studies in various locations such as the PM, plastid, MT and GA (SUBA database [26]). This cluster could be conceivably labeled as ER-localized, with 72% of the proteins known to localize to the ER.

Cluster 9 (Figure 5C, Table S7) from *L. multiflorum* contained 12 GA proteins as well as 5 ER, 2 plastid, 1 nuclear, 1 vacuolar, 5 PM and 24 unassigned proteins. The 12 GA proteins in this cluster are 6 vacuolar ATPases, 2 vacuolar sorting proteins, 3 Rab GTPases and an alpha SNAP protein, all of which are expected to reside in the TGN. The vacuolar sorting protein VSP45, a regulatory protein of the SNARE complex, is present in this cluster and has been shown to be localized in the GA using proteomics as well as immunocytochemistry [29]. Four of these proteins were also identified in the *P. trichocarpa* cluster 9 (Figure 5D, Table S8). This cluster contained the greatest number of GA proteins (4) compared to the other 12 clusters from *P. trichocarpa*, along with 2 PM proteins and 1 each from ER, MT, plastid and vacuole; the remaining 15 proteins were not assigned. Cluster 9 from both species appears to contain either TGN proteins or cargo proteins, although the profiles are slightly different (Figure 5C,D), with a higher enrichment in S1:S3 for *L. multiflorum* samples and S2:MM for *P. trichocarpa*.

In *L. multiflorum* the majority of the proteins depleted in S1:S3 and S1:MM are proteins expected to reside in the MT and plastid. The cluster with the most significant decreases, i.e., cluster 11 (Figure 5E), contained 42 MT, 17 plastid and 2 unknown proteins. The *P. trichocarpa* sample produced a cluster with a similar profile (Figure 5F) which contained 31 MT, 6 ER, 1 PM, 1 plastid, 1 vacuolar and 4 unknown proteins. In both species, cluster 11 could be considered as representing MT proteins.

## 4. Discussion

Although there have been considerable efforts made in defining the GA proteome in *Arabidopsis*, there are only two published studies in cereals (rice [11,12]) and 2 in conifers [13,14]). Here we provide a comparative analysis of the GA proteomes of a dicot (*P. trichocarpa*) and a commelinid monocot (*L. multiflorum*). For this purpose, we have used classical membrane fractionation procedures available in most laboratories (sucrose density gradient centrifugation). Profiling of the GA-enriched fractions (S1) in both species revealed the occurrence of known resident GA proteins and TGN proteins. The list of identified proteins comprised loosely associated and/or tethered proteins and transient or cargo proteins, all of which confirmed significant enrichment of intact GA. Interestingly, the GA-associated proteins that are present in the samples from both species, such as the coatomer complex proteins and RGP proteins, have not been identified in the *Arabidopsis* GA proteome [2].

Most noticeably absent from the *P. trichocarpa* S1 fraction were GTs involved in cell wall biosynthesis, possibly due to the lower level of GA enrichment in the samples prepared from this species. Although many expected GA proteins were present in the S1 fraction from both species, only 25% and 18% of the proteins in this fraction were assigned as GA proteins in *L. multiflorum* and *P. trichocarpa*, respectively. Similar limited levels of enrichments have been reported for other species [13]. Approximately 30% of the proteins in the GA-enriched fractions from *L. multiflorum* and *P. trichocarpa* were assigned to other organelles. As these data did not represent sufficient evidence for any biological conclusions, a relative quantitation approach was taken using the iTRAQ technology. 

Although the marker enzyme assays and the immunological characterization of the different fractions suggested that the S1 fraction was enriched in GA compared to all other fractions, this was not reflected in the S1:S2 ratio following sucrose density gradient fractionation. The cut-off chosen for enrichment in the iTRAQ analysis was 1.3, i.e., very similar to the fold observed (1.4) in the enzyme assays. When combined with the inherent problem of ratio suppression in the iTRAQ method [41], this lead to virtually no observed changes in GA proteins in S1 compared to S2. Thirty known GA proteins were identified in the *L. multiflorum* sample using the iTRAQ approach, with 17 of these proteins enriched in S1:S3 and S1:MM. In addition, enriched in both S1:S3 and S1:MM were proteins known to reside in other sub-cellular locations, i.e., 3 CP proteins, 8 ER proteins, 6 PM proteins, 2 nuclear proteins, 2 vacuolar proteins and 1 plastid protein. As the ER is closely associated to the GA and therefore difficult to separate, it is a common contaminant in GA preparations prepared by density centrifugation. This is reflected in the enrichment of 8 ER proteins in S1:S3 and S1:MM in *L. multiflorum*. To discriminate the GA proteins from others, *k*-means clustering was applied to the iTRAQ data, producing 13 clusters from the samples from both species. A potential GA cluster (cluster 13) and a potential ER cluster (cluster 12) was observed in *L. multiflorum*. No similar clusters were observed in *P. trichocarpa,* although potential TGN (cluster 9) and MT (cluster 11) clusters were observed.

Although the immunological analyses of the *P. trichocarpa* profile was similar to that of *L. multiflorum*, the “enrichment” was not reflected in the proteomic analysis. Thus, the iTRAQ analysis did give some insights into the GA proteome of *L. multiflorum* but not for that of *P. trichocarpa*. Whilst a similar distribution of sub-cellular locations of the identified proteins was observed the trends in iTRAQ ratios were not and neither were members of the most abundant GA protein family, the EMP70 detected. 

## 5. Conclusions

Protein profiling of the GA-enriched fractions allowed the identification of approximately 220 GA proteins from each species, but 30% of the proteins found in the GA-“enriched” fractions were considered to be contaminating proteins. The iTRAQ analyses only identified 83 potential GA proteins when cluster 13 (GA) and cluster 9 (TGN) are considered in *L. multiflorum* and 26 proteins in *P. tricocarpa* from cluster 9 (TGN). Although iTRAQ analysis is quantitative and hence gives more definitive localization information it is not as comprehensive as qualitative protein profiling due to the inherent limitations of the methodology. Development of methods allowing greater enrichment of the GA is needed in order to gain meaningful data from a protein profiling experiment. There is evidence to suggest that GA morphology can change during active secretion [42] and although we chose SCCs as a source of homogenous single cell type, it is known that the number of GA stacks and their size can vary within a single cell type [43]. For example, it is not clear whether all GA make and/or transport the same cargo proteins simultaneously [1] and as a consequence, the components in each individual GA could vary, contributing to different GA morphology/density/properties. Despite the evidence that GA is a highly dynamic organelle the enrichment techniques employed thus far, such as density gradients and FFE, have relied on the GA being morphologically homogeneous. Thus, a greater understanding of GA morphology, only possible through immuno-imaging approaches, would greatly enhance our ability to consider alternate techniques to purify GA. For example, using a series of antibodies to isolate various compartments of the GA, such as recently reported by Drakakaki et al. [29] to analyze the TGN, should be considered.

## Figures and Tables

**Table 1:** Enzymatic marker assays of fractions MM, S1, S2 and S3 from *L. multiflorum* SCCs.

Marker Activity	Membrane	MM	S1	S2	S3
IDPase (nmol/min/mg)	GA	1.04 (0.02)	1.70 (0.17)	1.22 (0.18)	0.47 (0.01)
Cyto.-*c* reductase (nmol/min/mg)	ER	280 (10)	200 (20)	240 (10)	340 (20)
Nitrate sensitive ATPase (nmol/min/mg)	Vacuole	66.24 (3.42)	39.25 (3.85)	137.26 (32.63)	178.51 (0.01)
Vanadate sensitive ATPase (nmol/min/mg)	PM	389.95 (13.51)	188.65 (13.87)	448.43 (2.32)	681.63 (46.81)
Cyto.-*c* oxidase (nmol/min/mg)	MT	2140 (1100)	210 (50)	720 (210)	2560 (590)

Enzyme activity is the average activity from three biological replicates. Numbers in parentheses indicate standard error.

**Table 2:** Proteins from cluster 13 (representing GA proteins) based on the iTRAQ analysis of *L. multiflorum* fractions.

Protein ID	TAIR10 ID	TAIR10 Description	Previous GA Proteomes	Function
LP_11479	AT3G19820.1	DWARF1	[10,29]	Cell elongation
p6229	AT4G12700.1	Unknown, contains a *O*-FucT-like domain		Non-classified glycosyltransferase
LP_2216	AT3G62830.1	UDP-glucuronic acid decarboxylase	[7,8,9,10]	Nucleotide sugar biosynthesis
p2877	AT1G30620.1	UDP-arabinose 4-epimerase 1	[9]	Nucleotide sugar biosynthesis
p20671	AT2G30500.1	kinase interacting family protein		Signaling
p4201	AT3G04080.1	apyrase 1	[7,8,9,10]	Transport
p3712	AT3G57330.1	autoinhibited Ca^2+^-ATPase 11	[9]	Transport
p45403	AT1G04120.1	multidrug resistance-associated protein 5		Transport
p42314	AT2G25520.1	putative sugar phosphate/phosphate translocator	[7,10]	Transport
p2354	AT4G35300.1	tonoplast monosaccharide transporter2	[9]	Transport
LP_6010	AT3G52850.1	Vacuolar Sorting Receptor-1 (VSR-1)	[8,9,10]	Transport
p4069	AT2G14740.1	vaculolar sorting receptor 3	[7,8,9]	Transport
p3235	AT4G39080.1	vacuolar proton ATPase A3	[9,10,29]	Transport
p3662	AT4G14240.1	CBS domain-containing protein with a DUF21		Unknown
p5363	AT5G10840.1	Endomembrane protein 70 protein	[6,7,8,9,10,29]	Unknown
p5735	AT5G35160.2	Endomembrane protein 70 protein	[9]	Unknown
LP_598	AT2G24170.1	Endomembrane protein 70 protein	[9]	Unknown
p820	AT3G13772.1	transmembrane nine 7	[6,7,8,9,10]	Unknown
p7705	AT5G51570.1	SPFH/Band 7/PHB domain-containing protein	[9,10]	Unknown
p2170	AT5G16250.1	unknown protein		Unknown
p9698	AT4G23790.1	unknown protein		Unknown
p50050		predicted protein		Unknown

## Supplementary Materials

The following are available online at www.mdpi.com/2227-7382/4/3/23/s1, Table S1: iTRAQ data of same-same trial to determine iTRAQ ratio cut-offs, Table S2: *L. multiflorum* protein profiling results of fraction S1, Table S3: *P. trichocarpa* protein profiling results of fraction S1, Table S4: *L. multiflorum* iTRAQ results, Table S5: *P. trichocarpa* iTRAQ results, Table S6: *L. multiflorum* cluster 12 (ER proteins), Table S7: *L. multiflorum* cluster 9 (TGN proteins), Table S8: *P. trichocarpa* cluster 9 (TGN proteins).

## Abbreviations

The following abbreviations are used in this manuscript:

ATTPS	trehalose phosphatase/synthase
BiP	luminal-binding protein
COG	oligomeric Golgi complex
COX	cytochrome-c oxidase
CP	cytoplasmic
CSL	cellulose synthase-like
DTT	dithiothreitol
EMP70	endomembrane 70 protein
ER	endoplasmic reticulum
FFE	Free Flow Electrophoresis
GA	Golgi apparatus
GC6	golgin candidate 6
GSL	glucan synthase-like
GT	glycosyltransferase
IDPase	inosine diphosphatase
iTRAQ	Isobaric tags for relative and absolute quantitation
LOPIT	Localization of Organelle Proteins by Isotope Tagging
MLG	β-1,3;1,4-glucans
MM	microsomal membrane
MMTS	methylmethane thiosulphate
MT	mitochondria
PM	plasma membrane
RGP	reversibly glycosylated protein
SAM	*S*-adenosyl-l-methionine-dependent methyltransferase
SCCs	suspension cell cultures
SNARE	*N*-ethylmaleimide-sensitive factor attachment protein receptors
TCEP	*tris-*(2-carboxyethyl) phosphine
TGN	*trans-*Golgi network
TRAPP	transport protein particle
UGE	UDP-d-glucose/UDP-d-galactose 4 epimerase
UGP	UDP-d-glucose pyrophosphorylase
VAMP	vesicle-associated membrane proteins
VSR	vacuolar sorting receptors
